# Data on the concentration of heavy metals and metalloids in lotic water of the Mantaro river watershed and human risk assessment, Peru

**DOI:** 10.1016/j.dib.2020.105493

**Published:** 2020-04-21

**Authors:** María Custodio, Richard Peñaloza, Ciro Espinoza, Tessy Peralta-Ortiz, Alberto Ordinola-Zapata, Héctor Sánchez-Suárez, Enedia Vieyra-Peña

**Affiliations:** a Facultad de Medicina Humana, Centro de Investigación de Medicina en Altura y Medio Ambiente, Universidad Nacional del Centro del Perú, Av. Mariscal Castilla N° 3909, Huancayo, Perú; b Facultad de Ingeniería Pesquera y Ciencias del Mar, Universidad Nacional de Tumbes, Calle Los Ceibos S/N, Puerto Pizarro, Tumbes, Perú; c Facultad de Ciencias Agrarias, Departamento Académico de Medicina Veterinaria y Zootecnia, Universidad Nacional de Tumbes, La Cruz S/N, Tumbes, Perú

**Keywords:** Lotic waters, Heavy metals, Metalloids, Human Risk

## Abstract

This article contains data on the concentration of heavy metals and metalloids in the water of seven rivers in the Mantaro river watershed in the central Andes of Peru, collected during the autumn of 2019. The concentrations of Cu, Fe, Pb, Zn and As were determined by flame atomic absorption spectrophotometry to assess human risk. The concentration of heavy metals and arsenic varied according to the sector of the rivers evaluated. The cluster analysis identified four different groups among the observation sectors. The risk assessment for humans was conducted on the basis of exposure doses to heavy metals and arsenic in water by ingestion and dermal pathways, using standard methods established by USEPA. These data can be reused as a basis for estimating the cancer risk or as a comparison group for future risk studies. They can also be useful to public health policy makers when proposing surveillance and control programmes using remedial technologies.

Specifications table

**Table:** 

Subject	Environmental Science
Specific subject area	Water quality and health risk assessment
Type of data	Tables and figures
How data was acquired	Analytical determinations of Cu, Fe, Pb, Zn and As by the method of atomic absorption spectrophotometry by flame, using a AA-6800 Atomic Absorption Spectrophotometer, Varian AA240 and standard methods.
Data format	Raw, analyzed
Parameters for data collection	Definition of sampling sectors, sample collection and digestion of the samples [1]. Determination of human risk from concentrations of heavy metals and arsenic in water.
Description of data collection	Two litres of water were collected in each sampling sector, in the opposite direction to the flow of the current at a depth of 20 cm, in autumn 2019. The transport and storage of the samples were carried out according to standard methods [2].
Data source location	Mantaro, Chia, Shullcas, Cunas, Chilca, Miraflores and Chancha rivers, located in the Andes Mountains, central region of Peru, between parallels 10°34’30” and 13°35’30” south latitude, and meridians 73°55’00” and 76°40’30” west longitude.
Data accessibility	Data is available in the article.

## Value of the data

• High concentrations of Pb and As in water can cause significant changes in organ systems. Therefore, it is urgent to control and reduce contamination levels.
• Data from analyses of heavy metals and metalloids in surface water in this area of study may be useful for public health policy makers in proposing monitoring and control programs through remedial technologies.
• These data can be used as a basis for estimating the carcinogenic risk or as baseline data for future risk studies of heavy metals and metalloids.
• The data could be used by authorities and policy makers to audit water quality.

## 1 Data description

### 1.1 Study area

**Fig. 1:**
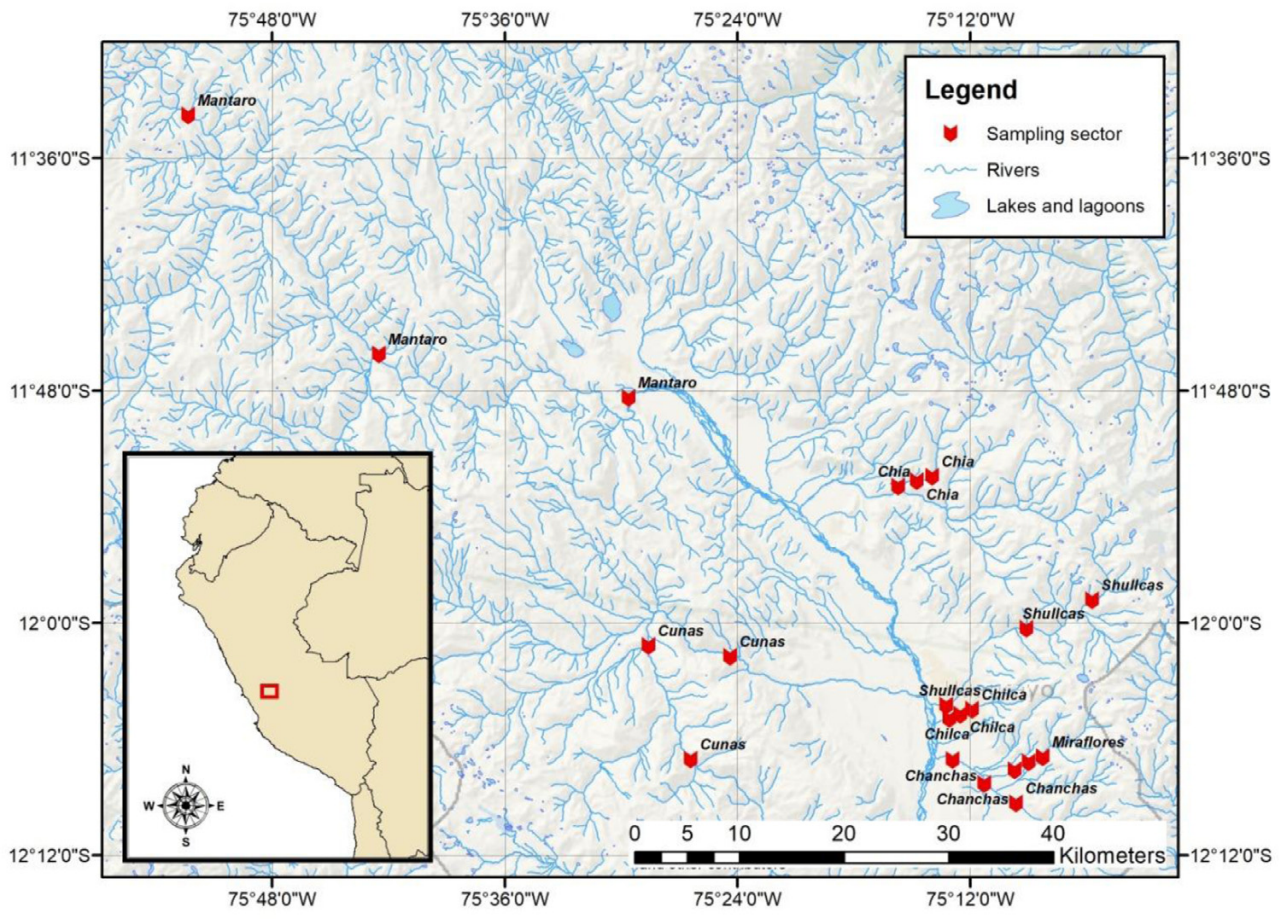
Location map of the study area in the Mantaro river watershed, Peru.

The rivers included in the study are located in the Mantaro River watershed located in the Andes Mountains of Peru, central region, between the parallels 10°34’30” and 13°35’30” south latitude, and the meridians 73°55’00” and 76°40’30” west longitude [3]. The Mantaro River is the main river of the watershed, it runs through areas with great mining influence, from the city of Cerro de Pasco to the Cobriza mine (located in the southeast of the basin). The Cunas River originates in the western mountain range at about 5,180 m above sea level. Its main course describes the shape of the letter S, with a west-east direction. The Shullcas River originates in the snowy Huaytapallana, and similarly to the Chia, Chilca, Miraflores and Chanchas rivers (Fig. 1), it experiences important water derivations for a variety of uses.

### 1.2 Analytical data

**Fig. 2:**
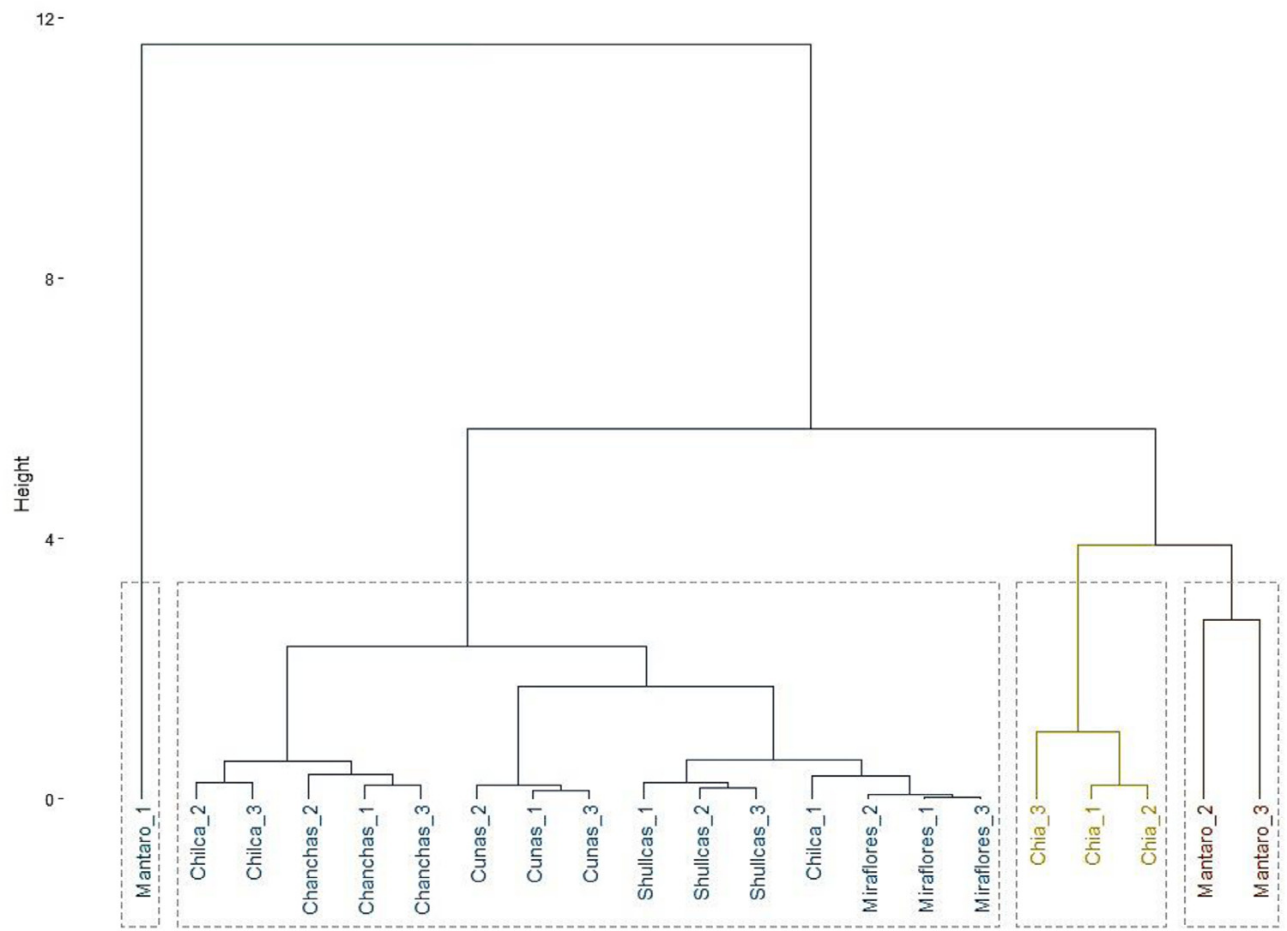
Distribution of observations and hierarchical clustering by river.

The data presented in this manuscript provide information on the concentration of Cu, Fe, Pb, Zn and As in the waters of seven rivers in the Mantaro River Basin and the ratios and indices to calculate the risk to human health [4,5]. Table 1 presents the data on the concentrations of heavy metals and arsenic detected in the waters of each of the sampling sectors of the rivers evaluated and Table 2 presents the average values ± SD. A cluster analysis by the Ward method (Fig. 2) was also carried out to classify the observations according to the degree of similarity and difference between the rivers evaluated [7].

**Table 1:** Concentration of heavy metals and arsenic in lotic waters of the Mantaro river watershed (µg/L).

River	Sampling sector	Cu	Pb	Zn	Fe	As
Mantaro	S1	21.6	20.0	90.7	2841.0	26.2
	S2	6.9	4.5	57.7	502.5	12.1
	S3	15.3	4.0	26.6	77.9	25.0
Cunas	S1	1.9	nd	9.3	9.5	9.0
	S2	1.7	nd	10.1	11.9	7.0
	S3	2.1	nd	8.5	7.1	8.0
Shullcas	S1	1.1	0.7	13.3	91.0	3.0
	S2	1.3	0.7	11.8	95.7	1.0
	S3	1.0	0.8	14.8	86.3	1.0
Chilca	S1	1.2	0.4	6.3	157.1	0.7
	S2	1.0	3.5	5.8	147.0	0.69
	S3	1.4	4.5	6.8	167.2	0.71
Miraflores	S1	1.7	nd	11.2	183.2	nd
	S2	1.8	nd	11.8	188.4	nd
	S3	1.6	nd	10.6	178.0	nd
Chía	S1	1.4	nd	15.3	14.4	14.0
	S2	1.4	nd	15.6	10.0	16.0
	S3	1.3	nd	15.0	18.8	23.0
Chanchas	S1	1.0	4.0	13.2	217.0	nd
	S2	8.7	3.9	16.7	145.0	nd
	S3	1.1	4.1	9.7	289.0	nd

nd: not detected.

**Table 2:** Mean and standard deviation of heavy metal and arsenic concentrations in the lotic waters of the Mantaro river watershed.

River	Cu	Pb	Zn	Fe	As
Mantaro	14.60 ± 7.37	9.50 ± 9.10	58.30 ± 32.10	1140* ± 1488.0	21.10* ± 7.82
Cunas	1.90 ± 0.20	nd	9.30 ± 0.80	9.50 ± 2.40	8.00 ± 1.00
Shullcas	1.13 ± 0.15	0.73 ± 0.06	13.30 ± 1.50	91.00 ± 4.70	1.67 ± 1.16
Cunas	1.90 ± 0.20	nd	9.30 ± 0.80	9.50 ± 2.40	8.00 ± 1.00
Chilca	1.20 ± 0.20	2.80 ± 2.14	6.30 ± 50	157.10 ± 10.10	0.70 ± 0.01
Miraflores	1.70 ± 0.10	nd	11.20 ± 0.60	183.20 ± 5.20	nd
Chía	1.37 ± 0.06	nd	15.30 ± 0.30	14.40 ± 4.40	17.67* ± 4.73

**Fig. 3:**
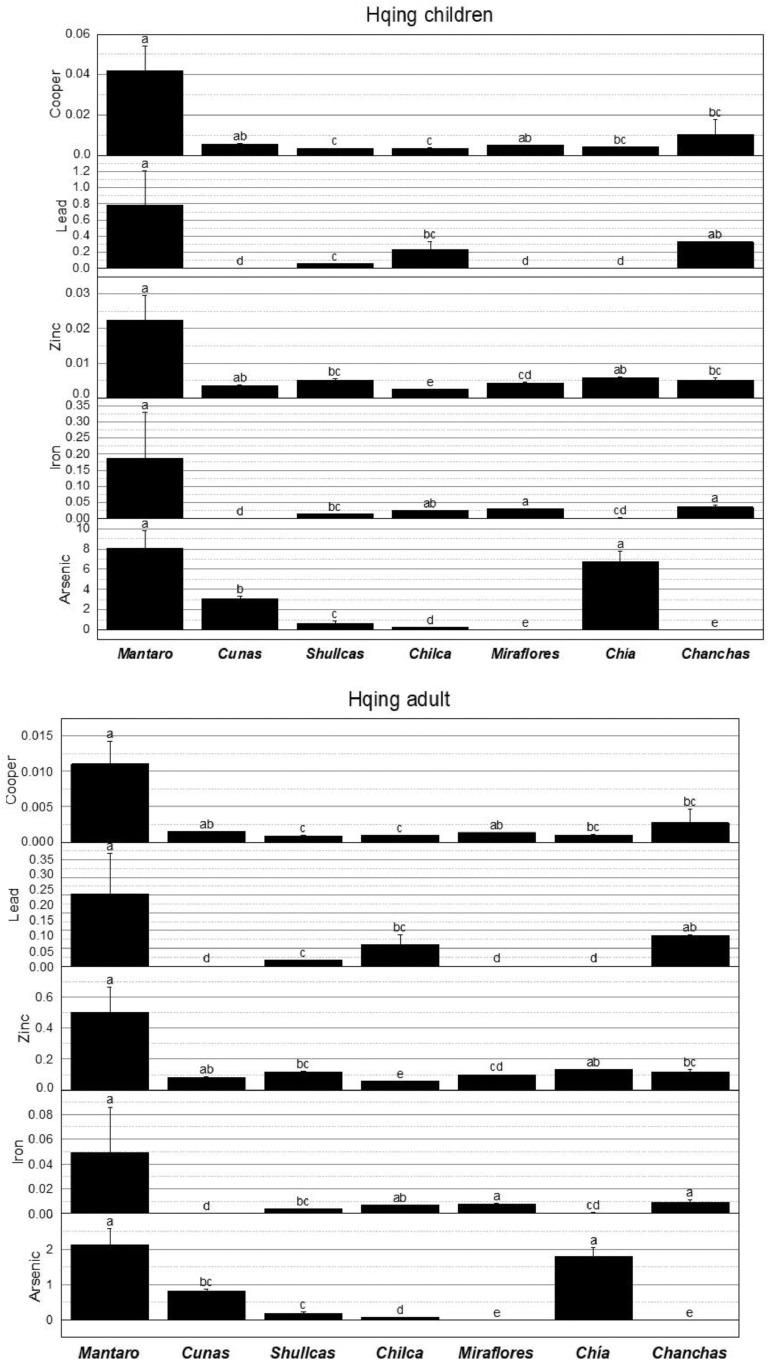
Kruskal–Wallis test for HQ_ing_ in children and adults by element and river evaluated.

Tables 3 and 4 show the exposure dose values via ingestion (D_ing_) and dermal (D_der_) of heavy metals and arsenic in children and adults in lotic waters with mining influence in the Mantaro River watershed. The obtained exposure dose values and the oral/dermal reference dose values (RfD_ing/der_) were used to calculate the risk quotient for ingestion (HQ_ing_) and dermal via (HQ_der_) of heavy metals and arsenic shown in Tables 5 and 6. In addition, Fig. 3 shows the Kruskal–Wallis test for HQing in children and adults by element and river evaluated. Table 7 shows the values of the hazard index for ingestion and dermal contact of heavy metals and arsenic in lotic waters with mining influence.

**Table 3:** Exposure dose values for ingestion (D_ing_) of heavy metals and arsenic in children and adults in lotic waters with mining influence in the Mantaro River Watershed, Peru.

River	Sampling sector	D_ing_ Cu	D_ing_ Cu	D_ing_ Pb	D_ing_ Pb	D_ing_ Zn	D_ing_ Zn	D_ing_ Fe	D_ing_ Fe	D_ing_ As	D_ing_ As
		Children	Adults	Children	Adults	Children	Adults	Children	Adults	Children	Adults
Mantaro	S1	2.485479449	0.650958904	2.30136986	0.602739726	10.4367123	2.73342466	326.909589	85.6191781	3.01479452	0.789589041
	S2	0.793972602	0.207945205	0.517808219	0.135616438	6.63945205	1.73890411	57.8219177	15.1438356	1.39232877	0.364657534
	S3	1.760547943	0.46109589	0.460273972	0.120547945	3.06082191	0.80164384	8.9638356	2.34767123	2.87671233	0.753424658
Cunas	S1	0.218630137	0.057260274	nd	nd	1.07013698	0.28027397	1.09315068	0.28630137	1.03561644	0.271232877
	S2	0.195616438	0.051232877	nd	nd	1.16219178	0.30438356	1.36931507	0.35863014	0.80547945	0.210958904
	S3	0.241643835	0.063287671	nd	nd	0.97808219	0.25616438	0.8169863	0.2139726	0.92054794	0.24109589
Shullcas	S1	0.126575342	0.033150685	0.080547945	0.02109589	1.53041096	0.40082192	10.4712329	2.74246575	0.34520548	0.090410959
	S2	0.149589041	0.039178082	0.080547945	0.02109589	1.35780822	0.35561644	11.0120548	2.88410959	0.11506849	0.030136986
	S3	0.115068493	0.030136986	0.092054794	0.024109589	1.7030137	0.4460274	9.93041095	2.60082192	0.11506849	0.030136986
Chilca	S1	0.138082192	0.036164384	0.046027397	0.012054795	0.72493151	0.18986301	18.0772603	4.73452055	0.08054795	0.02109589
	S2	0.115068493	0.030136986	0.402739726	0.105479452	0.66739726	0.17479452	16.9150685	4.43013699	0.07939726	0.020794521
	S3	0.16109589	0.042191781	0.517808219	0.135616438	0.78246575	0.20493151	19.239452	5.03890411	0.08169863	0.02139726
Miraflores	S1	0.195616438	0.051232877	nd	nd	1.28876712	0.33753425	21.0805479	5.52109589	nd	nd
	S2	0.207123287	0.054246575	nd	nd	1.35780822	0.35561644	21.6789041	5.67780822	nd	nd
	S3	0.184109589	0.048219178	nd	nd	1.21972603	0.31945205	20.4821918	5.36438356	nd	nd
Chía	S1	0.16109589	0.042191781	nd	nd	1.76054794	0.46109589	1.6569863	0.4339726	1.6109589	0.421917808
	S2	0.16109589	0.042191781	nd	nd	1.79506849	0.47013699	1.15068493	0.30136986	1.84109589	0.482191781
	S3	0.149589041	0.039178082	nd	nd	1.7260274	0.45205479	2.16328767	0.56657534	2.64657534	0.693150685
Chanchas	S1	0.115068493	0.030136986	0.460273972	0.120547945	1.51890411	0.39780822	24.969863	6.53972603	nd	nd
	S2	1.001095889	0.262191781	0.448767123	0.117534247	1.92164383	0.50328767	16.6849315	4.36986301	nd	nd
	S3	0.126575342	0.033150685	0.471780821	0.123561644	1.11616438	0.29232877	33.2547945	8.70958904	nd	nd

nd: not detected.

**Table 4:** Dermal exposure dose values (D_der_) for heavy metals and arsenic in children and adults in lotic waters with mining influence in the Mantaro River Watershed, Peru.

River	Sampling sector	D_der_ Cu	D_der_ Cu	D_der_ Pb	D_der_ Pb	D_der_ Zn	D_der_ Zn	D_der_ Fe	D_der_ Fe	D_der_ As	D_der_ As
		Children	Adults	Children	Adults	Children	Adults	Children	Adults	Children	Adults
Mantaro	S1	0.009113425	0.003089094	0.033753425	0.011441088	0.229607671	0.077828001	1.198668493	0.406301638	0.011054247	0.003746956
	S2	0.002911233	0.000986794	0.007594521	0.002574245	0.146067945	0.049511308	0.212013699	0.071864334	0.005105205	0.001730465
	S3	0.006455342	0.002188108	0.006750685	0.002288218	0.067338082	0.022824971	0.032867397	0.011140759	0.010547945	0.00357534
Cunas	S1	0.000801644	0.000271726	nd	nd	0.023543014	0.007980159	0.004008219	0.001358629	0.00379726	0.001287122
	S2	0.00071726	0.000243123	nd	nd	0.025568219	0.008666624	0.005020822	0.001701862	0.002953425	0.001001095
	S3	0.000886027	0.000300329	nd	nd	0.021517808	0.007293694	0.002995616	0.001015397	0.003375342	0.001144109
Shullcas	S1	0.00046411	0.000157315	0.00118137	0.000400438	0.033669041	0.011412485	0.038394521	0.013014238	0.001265753	0.000429041
	S2	0.000548493	0.000185918	0.00118137	0.000400438	0.029871781	0.010125363	0.040377534	0.013686402	0.000421918	0.000143014
	S3	0.000421918	0.000143014	0.001350137	0.000457644	0.037466301	0.012699608	0.036411507	0.012342074	0.000421918	0.000143014
Chilca	S1	0.000506301	0.000171616	0.000675068	0.000228822	0.015948493	0.005405914	0.066283288	0.022467437	0.000295342	0.00010011
	S2	0.000421918	0.000143014	0.005906849	0.00200219	0.01468274	0.004976873	0.062021918	0.021022999	0.000291123	9.86794E-05
	S3	0.000590685	0.000200219	0.007594521	0.002574245	0.017214247	0.005834955	0.070544657	0.023911874	0.000299562	0.00010154
Miraflores	S1	0.00071726	0.000243123	nd	nd	0.028352877	0.009610514	0.077295342	0.026200092	nd	nd
	S2	0.000759452	0.000257424	nd	nd	0.029871781	0.010125363	0.079489315	0.026943762	nd	nd
	S3	0.000675068	0.000228822	nd	nd	0.026833973	0.009095665	0.07510137	0.025456421	nd	nd
Chía	S1	0.000590685	0.000200219	nd	nd	0.038732055	0.013128648	0.006075616	0.002059396	0.005906849	0.00200219
	S2	0.000590685	0.000200219	nd	nd	0.039491507	0.013386073	0.004219178	0.001430136	0.006750685	0.002288218
	S3	0.000548493	0.000185918	nd	nd	0.037972603	0.012871224	0.007932055	0.002688656	0.00970411	0.003289313
Chanchas	S1	0.000421918	0.000143014	0.006750685	0.002288218	0.03341589	0.011326677	0.091556164	0.031033951	nd	nd
	S2	0.003670685	0.001244218	0.006581918	0.002231012	0.042276164	0.014329963	0.061178082	0.020736972	nd	nd
	S3	0.00046411	0.000157315	0.006919452	0.002345423	0.024555616	0.008323392	0.121934247	0.04133093	nd	nd

nd: not detected.

**Table 5:** Hazard quotient values for ingestion (HQ_ing_) of heavy metals and arsenic for children and adults in lotic waters with mining influence in the Mantaro River Watershed, Peru.

River	Sampling sector	HQ_ing_ Cu	HQ_ing_ Cu	HQ_ing_ Pb	HQ_ing_ Pb	HQ_ing_ Zn	HQ_ing_ Zn	HQ_ing_ Fe	HQ_ing_ Fe	HQ_ing_ As	HQ_ing_ As
		Children	Adults	Children	Adults	Children	Adults	Children	Adults	Children	Adults
Mantaro	S1	0.062137	0.016274	1.64384	0.43053	0.034789	0.780978	0.467014	0.122313	10.049315	2.631963
	S2	0.019849	0.005199	0.36986	0.09687	0.022132	0.496830	0.082603	0.021634	4.641096	1.215525
	S3	0.044014	0.011527	0.32877	0.08611	0.010203	0.229041	0.012805	0.003354	9.589041	2.511416
Cunas	S1	0.005466	0.001432	nd	nd	0.003567	0.080078	0.001562	0.000409	3.452055	0.904110
	S2	0.004890	0.001281	nd	nd	0.003874	0.086967	0.001956	0.000512	2.684932	0.703196
	S3	0.006041	0.001582	nd	nd	0.003260	0.073190	0.001167	0.000306	3.068493	0.803653
Shullcas	S1	0.003164	0.000829	0.05753	0.01507	0.005101	0.114521	0.014959	0.003918	1.150685	0.301370
	S2	0.003740	0.000979	0.05753	0.01507	0.004526	0.101605	0.015732	0.004120	0.383562	0.100457
	S3	0.002877	0.000753	0.06575	0.01722	0.005677	0.127436	0.014186	0.003715	0.383562	0.100457
Chilca	S1	0.003452	0.000904	0.03288	0.00861	0.002416	0.054247	0.025825	0.006764	0.268493	0.070320
	S2	0.002877	0.000753	0.28767	0.07534	0.002225	0.049941	0.024164	0.006329	0.264658	0.069315
	S3	0.004027	0.001055	0.36986	0.09687	0.002608	0.058552	0.027485	0.007198	0.272329	0.071324
Miraflores	S1	0.004890	0.001281	nd	nd	0.004296	0.096438	0.030115	0.007887	nd	nd
	S2	0.005178	0.001356	nd	nd	0.004526	0.101605	0.030970	0.008111	nd	nd
	S3	0.004603	0.001205	nd	nd	0.004066	0.091272	0.029260	0.007663	nd	nd
Chía	S1	0.004027	0.001055	nd	nd	0.005868	0.131742	0.002367	0.000620	5.369863	1.406393
	S2	0.004027	0.001055	nd	nd	0.005984	0.134325	0.001644	0.000431	6.136986	1.607306
	S3	0.003740	0.000979	nd	nd	0.005753	0.129159	0.003090	0.000809	8.821918	2.310502
Chanchas	S1	0.002877	0.000753	0.32877	0.08611	0.005063	0.113659	0.035671	0.009342	nd	nd
	S2	0.025027	0.006555	0.32055	0.08395	0.006405	0.143796	0.023836	0.006243	nd	nd
	S3	0.003164	0.000829	0.33699	0.08826	0.003721	0.083523	0.047507	0.012442	nd	nd

**Table 6:** Hazard quotient values for the dermal pathway (HQ_der_) of heavy metals and arsenic for children and adults in lotic waters with mining influence in the Mantaro River Watershed, Peru.

River	Sampling sector	HQ_der_ Cu	HQ_der_ Cu	HQ_der_ Pb	HQ_der_ Pb	HQ_der_ Zn	HQ_der_ Zn	HQ_der_ Fe	HQ_der_ Pb	HQ_der_ As	HQ_der_ As
		Children	Adults	Children	Adults	Children	Adults	Children	Adults	Children	Adults
Mantaro	S1	0.000380	0.000129	0.080365	0.027241	0.001913	0.000649	0.008562	0.002902	0.036847	0.012490
	S2	0.000121	0.000041	0.018082	0.006129	0.001217	0.000413	0.001514	0.000513	0.017017	0.005768
	S3	0.000269	0.000091	0.016073	0.005448	0.000561	0.000190	0.000235	0.000080	0.035160	0.011918
Cunas	S1	0.000033	0.000011	nd	nd	0.000196	0.000067	0.000029	0.000010	0.012658	0.004290
	S2	0.000030	0.000010	nd	nd	0.000213	0.000072	0.000036	0.000012	0.009845	0.003337
	S3	0.000037	0.000013	nd	nd	0.000179	0.000061	0.000021	0.000007	0.011251	0.003814
Shullcas	S1	0.000019	0.000007	0.002813	0.000953	0.000281	0.000095	0.000274	0.000093	0.004219	0.001430
	S2	0.000023	0.000008	0.002813	0.000953	0.000249	0.000084	0.000288	0.000098	0.001406	0.000477
	S3	0.000018	0.000006	0.003215	0.001090	0.000312	0.000106	0.000260	0.000088	0.001406	0.000477
Chilca	S1	0.000021	0.000007	0.001607	0.000545	0.000133	0.000045	0.000473	0.000160	0.000984	0.000334
	S2	0.000018	0.000006	0.014064	0.004767	0.000122	0.000041	0.000443	0.000150	0.000970	0.000329
	S3	0.000025	0.000008	0.018082	0.006129	0.000143	0.000049	0.000504	0.000171	0.000999	0.000338
Miraflores	S1	0.000030	0.000010	nd	nd	0.000236	0.000080	0.000552	0.000187	nd	nd
	S2	0.000032	0.000011	nd	nd	0.000249	0.000084	0.000568	0.000192	nd	nd
	S3	0.000028	0.000010	nd	nd	0.000224	0.000076	0.000536	0.000182	nd	nd
Chía	S1	0.000025	0.000008	nd	nd	0.000323	0.000109	0.000043	0.000015	0.019689	0.006674
	S2	0.000025	0.000008	nd	nd	0.000329	0.000112	0.000030	0.000010	0.022502	0.007627
	S3	0.000023	0.000008	nd	nd	0.000316	0.000107	0.000057	0.000019	0.032347	0.010964
Chanchas	S1	0.000018	0.000006	0.016073	0.005448	0.000278	0.000094	0.000654	0.000222	nd	nd
	S2	0.000153	0.000052	0.015671	0.005312	0.000352	0.000119	0.000437	0.000148	nd	nd
	S3	0.000019	0.000007	0.016475	0.005584	0.000205	0.000069	0.000871	0.000295	nd	nd

nd: not detected.

**Table 7:** Hazard index for ingestion and dermal contact of heavy metals and arsenic in lotic waters with mining influence in the Mantaro River Watershed, Peru.

River	Sampling sector	HI_ing_	HI_ing_	HI_der_	HI_der_
		Children	Adults	Children	Adults
Mantaro	S1	12.257095	3.982058	0.128067	0.043411
	S2	5.13554	1.830859	0.037951	0.012864
	S3	9.984833	2.829921	0.052298	0.017727
Cunas	S1	3.46265	0.984597	0.012916	0.004378
	S2	2.695652	0.790675	0.010124	0.003431
	S3	3.078961	0.877149	0.011488	0.003895
Shullcas	S1	1.231439	0.434879	0.007606	0.002578
	S2	0.46509	0.221252	0.004779	0.00162
	S3	0.472052	0.248828	0.005211	0.001767
Chilca	S1	0.333066	0.139941	0.003218	0.001091
	S2	0.581594	0.200925	0.015617	0.005293
	S3	0.676309	0.233944	0.019753	0.006695
Miraflores	S1	0.039301	0.104325	0.000818	0.000277
	S2	0.040674	0.109716	0.000849	0.000287
	S3	0.037929	0.098935	0.000788	0.000268
Chía	S1	5.382125	1.538755	0.02008	0.006806
	S2	6.148641	1.742062	0.022886	0.007757
	S3	8.834501	2.44047	0.032743	0.011098
Chanchas	S1	0.372381	0.209111	0.017023	0.00577
	S2	0.375818	0.233989	0.016613	0.005631
	S3	0.391382	0.184225	0.01757	0.005955

## 2 Experimental design, materials and methods

The sampling was carried out in established sectors in the Mantaro, Chía, Shullcas, Cunas, Chilca, Miraflores and Chanchas rivers in the Junín region in the autumn of 2019. Water samples were taken in triplicate in each sampling sector in the opposite direction of the flow of the stream at a depth of 20 cm, one meter from the edge of each river [6]. The samples were conditioned in a cold chain and transported to the laboratory. The concentrations of Cu, Fe, Pb, Zn and As were determined by the method of flame atomic absorption spectrophotometry, according to the methodology recommended by the FAO (1983). using an AA-6800, Varian AA240 atomic absorption spectrophotometer. Previously, the calibration curve was prepared with standard solutions for Cu, Fe, Pb, Zn and As, supplied by Merck with a purity level of 99.98%. Finally, the calibration curve and the concentration of the samples were obtained.

The human risk assessment for exposure to heavy metals and arsenic in water via ingestion and dermal exposure [8,9] was calculated using the following equations:

(1) $$D_{ing}=\frac{C_{agua\;}\times IngR\times EF\times ED}{BW\times AT}$$

(2) $$D_{der}=\frac{C_{agua\;}\times SA\times KP\times ET\times EF\times ED\times CF}{BW\times AT}$$

The coefficient of dermal permeability for Cu, Pb, Zn, Fe and As is given as 0.001, 0.004, 0.006, 0.001 and 0.001 [10].

(3) $$HQ_{ing/der}=D_{ing/der}/RfD_{ing/der}$$

The oral and dermal reference dose (RfD_ing/der_) has been obtained from the literature [11].

(4) $$\text{HI}=\sum_{i=1}^{n}HQ_{\text{ing}/\text{der}}$$

## Appendix Supplementary materials

Image, application 1
